# Supplementary material for: Modeling glioblastoma heterogeneity as a dynamic network of cell states
Source: Mol Syst Biol. 2021 Sep 16;17(9):e10105. doi: 10.15252/msb.202010105 (PMC8444284; doi:10.15252/msb.202010105)
Supplement: Supplementary file 6 — Source Data for Figure 5 [file MSB-17-e10105-s004.zip › Figure5A_sourcedata/GSEA_3071/hallmarks_stateI.GseaPreranked.1623742531165/HALLMARK_MYC_TARGETS_V2.html]

Details for gene set HALLMARK\_MYC\_TARGETS\_V2[GSEA]

|  || Dataset | state13071 |
| Phenotype | NoPhenotypeAvailable |
| Upregulated in class | na\_neg |
| GeneSet | HALLMARK\_MYC\_TARGETS\_V2 |
| Enrichment Score (ES) | -0.44092157 |
| Normalized Enrichment Score (NES) | -1.5760748 |
| Nominal p-value | 0.06065574 |
| FDR q-value | 0.06704547 |
| FWER p-Value | 0.603 |
Table: GSEA Results Summary

  

Fig 1: Enrichment plot: HALLMARK\_MYC\_TARGETS\_V2      
 Profile of the Running ES Score & Positions of GeneSet Members on the Rank Ordered List

  

| PROBE | GENE SYMBOL | GENE\_TITLE | RANK IN GENE LIST | RANK METRIC SCORE | RUNNING ES | CORE ENRICHMENT || 1 | IMP4 |  |  | 1472 | -0.254 | -0.3944 | No |
| 2 | BYSL |  |  | 1503 | -0.257 | -0.3754 | No |
| 3 | PPAN |  |  | 1568 | -0.263 | -0.3654 | No |
| 4 | WDR43 |  |  | 1679 | -0.277 | -0.3672 | No |
| 5 | LAS1L |  |  | 1826 | -0.297 | -0.3771 | No |
| 6 | FARSA |  |  | 1932 | -0.310 | -0.3739 | No |
| 7 | PLK4 |  |  | 2167 | -0.346 | -0.4037 | Yes |
| 8 | PLK1 |  |  | 2206 | -0.353 | -0.3766 | Yes |
| 9 | TCOF1 |  |  | 2210 | -0.354 | -0.3394 | Yes |
| 10 | EXOSC5 |  |  | 2297 | -0.372 | -0.3241 | Yes |
| 11 | NOP56 |  |  | 2333 | -0.380 | -0.2933 | Yes |
| 12 | GNL3 |  |  | 2342 | -0.382 | -0.2545 | Yes |
| 13 | RRP9 |  |  | 2373 | -0.388 | -0.2214 | Yes |
| 14 | MRTO4 |  |  | 2393 | -0.391 | -0.1848 | Yes |
| 15 | HSPD1 |  |  | 2396 | -0.392 | -0.1433 | Yes |
| 16 | SRM |  |  | 2568 | -0.432 | -0.1459 | Yes |
| 17 | DCTPP1 |  |  | 2644 | -0.451 | -0.1189 | Yes |
| 18 | UNG |  |  | 2758 | -0.489 | -0.0988 | Yes |
| 19 | TMEM97 |  |  | 2834 | -0.514 | -0.0650 | Yes |
| 20 | PA2G4 |  |  | 3116 | -0.690 | -0.0714 | Yes |
| 21 | MCM5 |  |  | 3132 | -0.706 | 0.0002 | Yes |
| 22 | MCM4 |  |  | 3341 | -1.006 | 0.0487 | Yes |
Table: GSEA details [plain text format]

  

Fig 2: HALLMARK\_MYC\_TARGETS\_V2: Random ES distribution      
 Gene set null distribution of ES for **HALLMARK\_MYC\_TARGETS\_V2**
